# Spectral variation of fluorescence lifetime near single metal nanoparticles

**DOI:** 10.1038/srep21349

**Published:** 2016-02-15

**Authors:** Jia Li, Alexey V. Krasavin, Linden Webster, Paulina Segovia, Anatoly V. Zayats, David Richards

**Affiliations:** 1 Department of Physics, King’s College London, Strand, London WC2R 2LS, UK

## Abstract

We explore the spectral dependence of fluorescence enhancement and the associated lifetime modification of fluorescent molecules coupled to single metal nanoparticles. Fluorescence lifetime imaging microscopy and single-particle dark-field spectroscopy are combined to correlate the dependence of fluorescence lifetime reduction on the spectral overlap between the fluorescence emission and the localised surface plasmon (LSP) spectra of individual gold nanoparticles. A maximum lifetime reduction is observed when the fluorescence and LSP resonances coincide, with good agreement provided by numerical simulations. The explicit comparison between experiment and simulation, that we obtain, offers an insight into the spectral engineering of LSP mediated fluorescence and may lead to optimized application in sensing and biomedicine.

The pioneering work of Purcell^1^ identified that the intensity and lifetime of spontaneous emission are determined not only by the intrinsic characteristics of the emitter, but also by the material environment in which the emitter is located, or in other words the local electromagnetic density of states. In particular, when emitters are placed near a metallic nanostructure, the emission intensity can be drastically enhanced, accompanied by a reduction of emission lifetime^2^,^3^,^4^,^5^,^6^. This phenomenon, referred to as metal-enhanced fluorescence (MEF), is attributed to the excitation of plasmonic modes within the nanostructure. This allows a metallic nanoparticle to act as a resonant nano-antenna, modifying emission by enhancing light absorption by nearby emitters (excitation enhancement) accompanied by a change in their effective quantum yield by altering their radiative and non-radiative decay rates (emission enhancement and quenching).

There has been significant interest recently in improving the performance of existing fluorophores through incorporation with metallic nanostructures, motivated by potential applications ranging from plasmon-enhanced bioassays^7^,^8^,^9^,^10^ to novel optoelectronic devices^11^,^12^,^13^,^14^. To enable more efficient localized surface plasmon resonance (LSPR) related application, a variety of nanostructures have been studied, such as nanorods^15^, nanoshells^16^, silver island films^17^ and silver nanoprisms^18^,^19^ to derive a more complete picture of the role of the relative spectral overlap of excitation, LSPR and emission wavelengths on fluorescence intensity enhancement and lifetime reduction, as well as emitter non-radiative line broadening^20^.

The MEF process can be characterised both by the enhancement of fluorescence emission intensity, and by the modification of the fluorescence lifetime. The measurement of lifetime, in particular, is usually insensitive to variations in the number of fluorophores probed, excitation intensity, collection efficiency and photo-bleaching, all of which can affect intensity-based measurements. However, measurement of fluorescence lifetime for large ensembles of plasmonic nanoparticles results in inhomogeneous broadening of the LSPR. To derive a correlation between the emission spectra of fluorophores and the LSPR spectrum requires, instead, the use of single-particle spectroscopy applied to a population of plasmonic nanoparticles in the vicinity of fluorophores^18^,^19^. Here we demonstrate, at the single nanoparticle level, the dependence of MEF lifetime reduction on the spectral overlap between dye molecule fluorescence emission and the LSPR of spherical single gold nanoparticles (GNPs), surrounded by a large number of fluorescent molecules, averaging over all orientations and separations. The inherent nanoparticle inhomogeneity provides access to a range of LSP wavelengths, allowing the detailed study of spectral dependence of MEF. In contrast to previous single-particle lifetime studies of silver nanoprisms^19^, in which a wide range of LSPR resonance was achieved through the variation of nanoparticle shape, the more controlled restriction of nanoparticle geometry in the present work to spherical nanoparticles provides the opportunity to perform a direct comparison between experimental measurements and numerical simulation, to provide an in-depth understanding of the effect of spectral overlap on fluorescence modification.

## Results and Discussion

**Figure 1:**
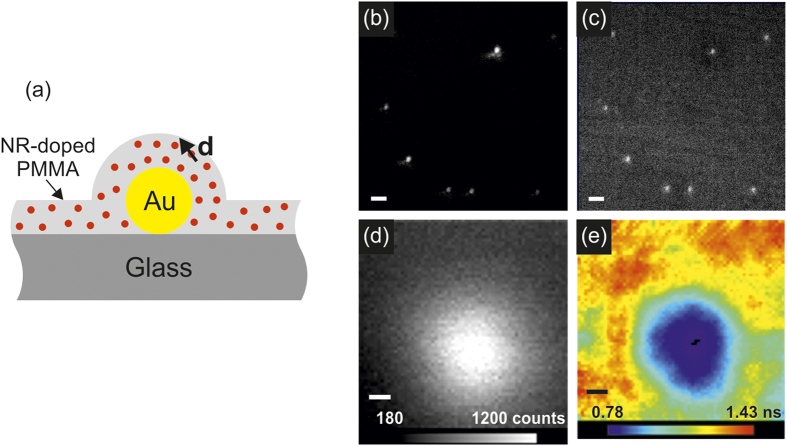
Sample layout and experimentally measured dark-field and fluorescence maps. (**a**) Sketch of the sample cross-section. (**b**) Dark-field light scattering image of GNPs embedded in a Nile Red-doped PMMA film and (**c**) the respective fluorescence intensity distribution; scale bars are 2 μm. (**d**) Fluorescence intensity and (**e**) LSPR modified lifetime of Nile Red in the vicinity of a single GNP; scale bars are 100 nm.

We have used fluorescence lifetime imaging microscopy (FLIM) to provide high-resolution spatial and temporal lifetime imaging of fluorescent molecules in the vicinity of GNPs, and correlated these measurements with the LSPR of the GNPs characterized by dark field spectroscopy. Isolated single 100 nm diameter GNPs embedded in a thin PMMA film doped with Nile Red molecules have been investigated (Fig. 1(a)). Wide-field dark field microscopy was used to identify the locations of single GNPs, while fluorescence microscopy identifies that Nile Red emission is enhanced in the vicinity of each GNP, with a one-to-one correspondence (Fig. 1(b,c)). For each GNP identified, dark field spectroscopy has been used to characterise the LSPR spectrum, while FLIM of 1 μm^2^ areas around each GNP was employed to determine the modification of the Nile Red fluorescence intensity and lifetime, averaged over the diffraction-limited spot of the measurement (Fig. 1(d,e)). It is immediately evident from the correlation of the fluorescence intensity distribution and nanoparticle position that the presence of the GNPs leads both to an enhancement in fluorescence emission from Nile Red molecules and a reduction in its lifetime.

Fluorescence intensity and lifetime is expected to display a strong dependence on both distance and orientation for dye molecules within the order of 100 nm from a nanoparticle^2^,^4^, and so these images, which average over a large number of dye molecules within the diffraction-limited spot of the measurement, provide a simple signature of the MEF process. Theoretically, the fluorescence signal time-dependence at the location of the GNP is given by


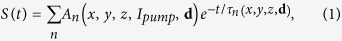


where 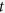 is the time after the excitation^21^. The summation is performed over all the molecules within the detection region, with the coefficient *A*_*n*_ characterising the contribution to the far-field radiation of each molecule, with the time dependence given by its fluorescence lifetime *τ*_*n*_. Both *A*_*n*_ and *τ*_*n*_ depend on the molecule’s position and dipole moment 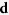, while additionally, 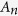 depends on the *local* excitation intensity 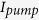. Within the concept of the average MEF lifetime, Eq. 1 can be represented as


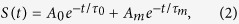
where 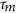 is the position- and orientation- averaged lifetime of fluorescence from molecules in close proximity to the GNP, experiencing strong modified emission, while 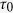 is the Nile Red lifetime for molecules whose fluorescence decay is unmodified (far away from the nanoparticle). In this way 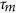 provides a simple parameterisation of the modification induced by the GNP of fluorescence lifetime, enabling straightforward comparison between different nanoparticles and with theory. 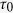 is determined to be 3.8 ns from single exponential lifetime fits from nearby sample regions containing no GNPs. This expression was found to provide a good fit to the experimental data (see Methods section for more details).

**Figure 2:**
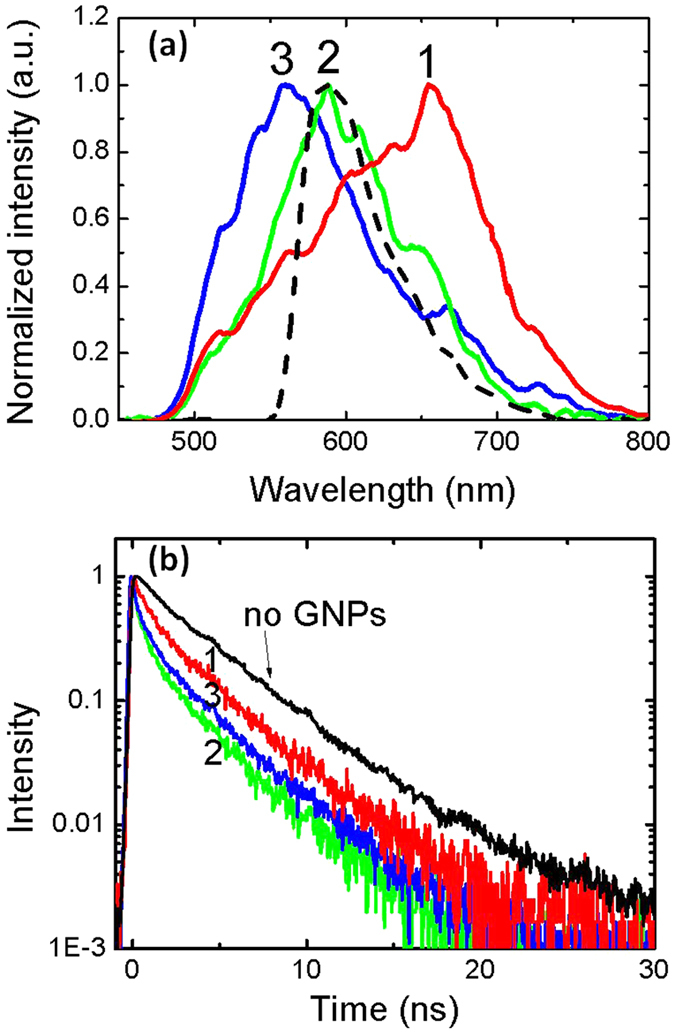
Experimental spectral and time dependencies. (**a**) Dark field scattering spectra from three single GNPs (solid lines) and fluorescence spectrum of the Nile Red-doped PMMA film (dashed line). (**b**) Fluorescence decays of Nile Red at the locations of the GNPs in (**a**); the decay for a reference sample is also plotted for comparison (black line). The strong red-shift identified in LSPR spectrum 1, and its associated longer fluorescence decay, represent a rare outlier measurement, which might result from a small nanoparticle aggregate or a rare non-spherical nanoparticle.

**Figure 3:**
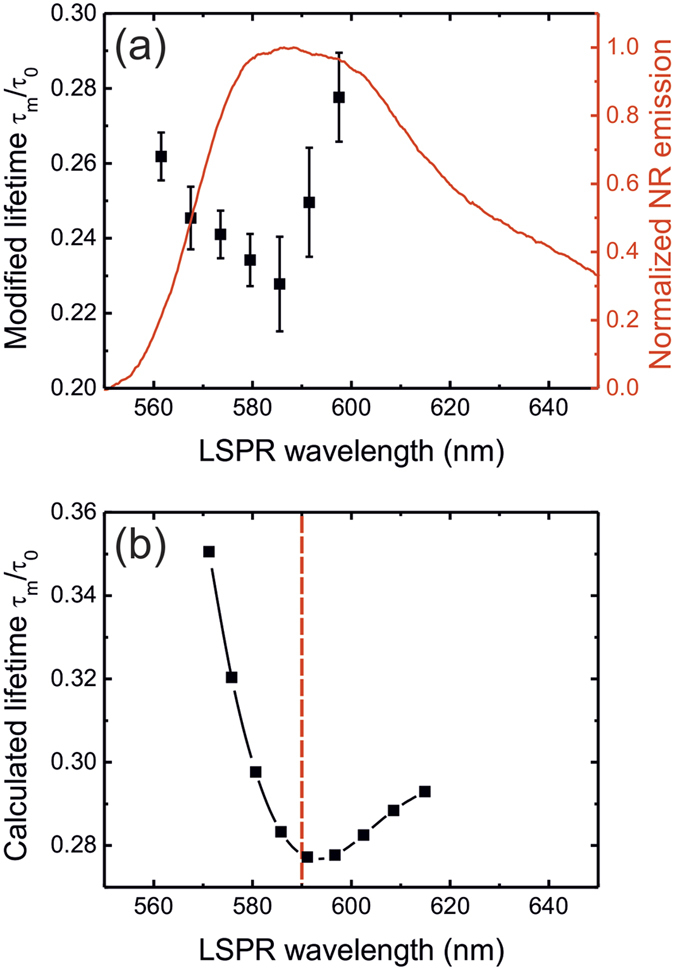
Lifetime modification in the presence of resonant GNPs. (**a**) The fluorescence lifetime reduction *τ*_*m*_/*τ*_0_ as a function of LSPR peak position; the Nile Red fluorescence spectrum from a thin film is also shown (red line). LSPR peak positions are binned together by 6 nm intervals, with the average value of the modified lifetime plotted for each bin (error bars show the standard deviation for each bin). (**b**) The calculated modified lifetime shows a strong dependence on the spectral overlap between LSPR and Nile Red emission (dashed line denotes the emission wavelength of the radiating dipole used in the simulations).

To explore the effect of spectral overlap on the fluorescence lifetime reduction, we have acquired lifetime data and corresponding LSP spectra from 45 single GNPs with varying LSPR frequencies, measured from different regions across the dye-doped film. Figure 2 shows three representative LSPR dark-field scattering spectra of single GNPs and the corresponding fluorescence decay curves determined at the respective GNP locations. A variation of nanoparticle size from the nominal 100 nm diameter, possible asymmetry of the nominally spherical nanoparticles, and also possible changes in the surrounding PMMA films, lead to variations in LSPR wavelength. Fluorescence decays measured in the vicinity of GNPs all exhibit a shorter lifetime, in contrast to a reference sample without GNPs. In particular, improved spectral overlap between the Nile Red emission and the LSPR leads to a faster fluorescence decay; the greatest lifetime reduction is observed when the nanoparticle LSP and fluorescence emission spectra coincide (Fig. 3(a)).

These observations can be explained by considering that the lifetime modification (the Purcell effect) is defined by the change in the local electromagnetic density of states 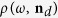 available for spontaneous emission, compared to that in free space, so that for inhomogeneously broadened fluorescence the emission enhancement is determined by the spectral overlap between the emission spectral lineshape and the density of states^22^,^23^:


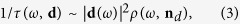


where 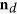 is a unit vector defining the orientation of the dipole moment 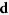.

This spectral dependence of the MEF lifetime modification was explored theoretically using finite element numerical simulations employing a simple illustrative model, in which a core-shell (CS-GNP) structure is adopted to mimic the experimental configuration, with the core diameter and the plasma frequency of gold varied to provide the range of experimentally observed LSPR wavelengths (see Methods for more details). All the decay channels were taken into account in the simulations: radiation into photons, coupling to LSPRs, non-radiative quenching and internal non-radiative decays. Non-radiative quenching plays a dominant role for distances between the emitter and the particle surface smaller than ~10 nm, while at larger distances the resonant coupling of the fluorophore emission to the LSPRs is well pronounced. The ratio between the radiative and non-radiative decay channels (both dependent on dipole position and orientation) also defines the local quantum efficiency of the emitter, or in other words its relative contribution to the signal measured in the far field, which was taken into account (see Methods for more details). Averaging the decay rates over all dipole positions and orientations in the PMMA shell, we show in Fig. 3(b) the reduction in lifetime calculated for a radiating dipole with a frequency corresponding to the peak of Nile Red fluorescence (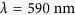). It can be seen that when the LSPR and Nile Red emission coincide, the lifetime shows the maximum reduction, providing good agreement with the experiment. In addition to the limitation of our model through the treatment of the fluorescent molecules as simple mono-energetic radiating dipoles, it is expected that a more accurate quantitative description of the observed lifetime modification can be achieved by further reducing the discrepancies between the experiment and the theoretical model, such as the role of the substrate as a reflective mirror, as well as the introduction of more complex profile of the PMMA coating, which are believed to considerably affect the fluorescence lifetime.

## Conclusion

Through a single nanoparticle investigation we have demonstrated that the reduction in fluorescence lifetime for fluorophores in close proximity to metal nanoparticles is strongly dependent on the spectral overlap between the fluorescence emission spectrum and the nanoparticle LSPR. A minimum fluorescence lifetime is observed when the peak for the dark-field LSPR scattering spectrum matches the maximum fluorescence emission wavelength, resulting from a significant modification of local photonic mode density and hence increase in the radiative decay rate. We note that this is in agreement with the results of ref. 18 and generally confirms the theoretical predictions of ref. 24. We find that tuning the LSPR to the emission maximises the coupling of the fluorophore excited state to the LSPR and therefore minimises the decay lifetime, with a commensurate enhancement in fluorescence signal also observed. In particular, our experimental observations have shown a good agreement with finite element numerical simulations, enabled through the simple geometry of the system under investigation. The principle of spectral-overlap dependent fluorescence modifications may allow control not only over the emission rates but also relative enhancement of the emission at a pre-selected wavelength, within the emission profile, by tuning the LSPRs, which maybe especially advantageous when the fluorescence linewidth is comparable or greater than the LSPR spectral width. These results pave the way for the design and engineering of the metal enhanced fluorescence decay process, particularly for the development of novel nanoscale sensors and biomedical devices.

## Methods

### Sample preparation

In order to immobilize single GNPs homogeneously on the substrate and to deposit GNPs, 0.2 mm glass coverslips (Agar Scientific, Stansted, UK) were pre-treated with a standard hydrophilic procedure^25^. The coverslips were sonicated in Decon 90 (Decon Laboratories, East Sussex, UK) for 3 hours, rinsed thoroughly in deionized water, and then immersed in an acid piranha solution for an hour. Slides for GNPs were also then immersed in base piranha solution for a further hour.

**Figure 4:**
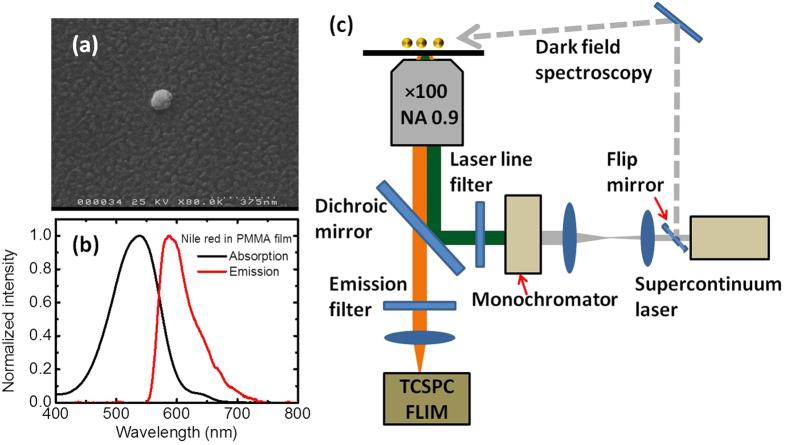
Samples and the experimental setup. (**a**) SEM image of single GNP on a glass coverslip. (**b**) Absorption and emission spectra of a Nile Red-doped PMMA film. (**c**) Schematic of FLIM and dark-field experimental setup. (The schematic was drawn by J.L.).

A distribution of single 100 nm diameter GNPs (BBI Gold Colloid, 8% maximum coefficient of variation in nanoparticle size) was realised by spin-coating the aqueous GNP solution on the hydrophilic glass coverslip. Scanning electron microscopy (SEM) of samples, coated with an additional 5 nm layer of gold by thermal evaporation, confirmed the presence of isolated nanoparticles (rather than aggregates), as shown in Fig. 4(a). Ten individual nanoparticles were imaged, all of which were identified by SEM as being single nanoparticles. A fluorophore-doped polymer solution was prepared by mixing Nile Red (~20 μM) and PMMA chloroform solutions together. A Nile Red-doped PMMA film was then spin-coated on the substrate with the single GNPs. The thickness of the PMMA film was measured to be 30 nm by AFM of a scratch in the film. The absorption and fluorescence emission spectra from the Nile Red-doped PMMA are shown in Fig. 4(b).

### Fluorescence and dark-field imaging and spectroscopy

Measurements were performed with an inverted microscope and 100 × air objective lens (0.9 NA) (Nikon Corporation, Japan) and sample scanning with a piezo-flexure stage, shown in Fig. 4(c). A supercontinuum laser (SC-450-2; Fianium, UK) with a repetition rate of 20 MHz and pulse duration of 400 fs was used as the light source, both for fluorescence excitation and dark field light scattering microscopy and spectroscopy.

Dark field measurements were performed by obliquely illuminating the sample surface from above with a very large incident angle. This allowed the collection of the scattered light from single GNPs while eliminating the direct illumination light from the laser source. Wide-field imaging of the scattered light with a CCD camera enabled the precise location of singe GNPs. For dark-field spectroscopy the scattered light was imaged onto the core of a 365 μm optical fibre, corresponding to a detection area on the sample of approximately 3 μm diameter. Light scattering spectra were then obtained using a fibre-coupled spectrometer (AvaSpec-2048, Avantes, The Netherlands).

For FLIM the supercontinuum output was passed through a prism monochromator and 514 nm laser line filter to determine the excitation wavelength. The full-width at half maximum of the diffraction limited focussed spot on the sample was ~300 nm. Excitation and emission collection was with a dichroic mirror and a long-pass 538 nm emission filter. Photon detection was with an avalanche photodiode (Id-100; Id Quantique, Switzerland) and a synchronous time-correlated single photon counting (TCSPC) module (SPC-150; Becker & Hickl, Germany). High resolution FLIM of 1 μm^2^ areas, each containing an individual GNP, were performed with 14 nm steps.

### Fluorescence lifetime analysis

**Figure 5:**
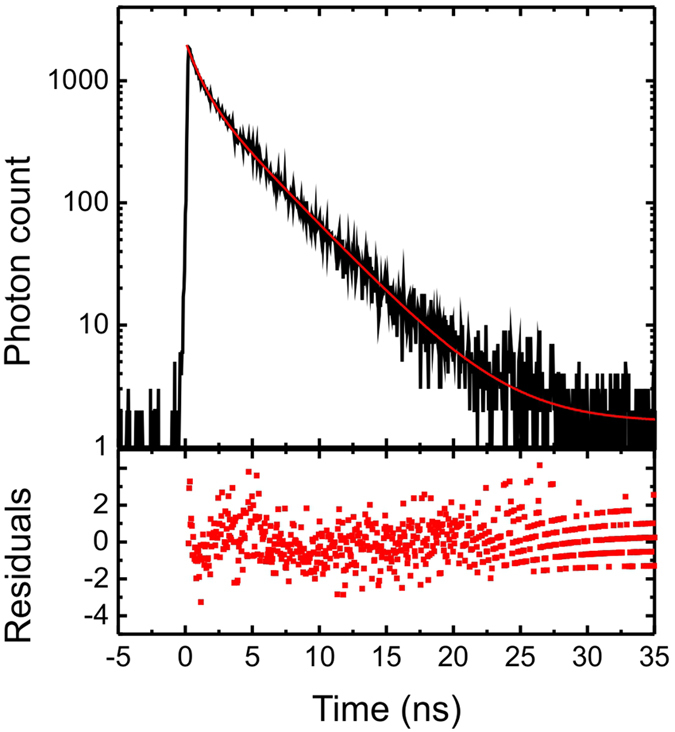
Fluorescence lifetime analysis. Top: Typical fluorescence decay fitted with a bi-exponential curve (*A*0 = 916, *τ*_0_ = 3.8 ns, *A*_*m*_ = 1186 and *τ*_*m*_ = 0.98 ns in the notations of Eq. 2). Bottom: residuals from the fitting.

Decay transients were fitted using iterative reconvolution software (TRI2)^26^, after correcting for background fluorescence and an instrument response time (between 120 and 180 ps). A centroiding algorithm was applied to the high resolution images of enhanced fluorescence in the vicinity of single GNPs, to determine the location of each GNP. A representative fluorescence decay was then determined for each GNP by binning a 7-pixel diameter circle (corresponding to a diameter of ~100 nm) centred on the GNP location, ensuring that the diffraction-limited spot of the measurement encompassed the nanoparticle for all data points. This was found to give consistent datasets of lifetime from different ensembles of nanoparticles. Decays were fitted with a bi-exponential decay, Eq. 2 (Fig. 5).

### Numerical simulations

Numerical studies were performed using COMSOL Multiphysics software. As a first step, in order to identify the parameters of the core-shell nanoparticles (CS-GNPs) corresponding to the observed LSPRs, spectral dependence of scattering of plane electromagnetic waves on the core-shell systems was investigated. The range of LSPR wavelengths observed in experiment results from a number of factors including both size and shape of the GNPs and small changes in their dielectric environment. Rather than attempting to simulate these effects, account was made of the full range of LSPR wavelengths observed by simply varying the radius of the metallic core of CS-GNP and the gold bulk plasma frequency. A Drude model with the addition of two Lorenz contributions, fitting Johnson and Christy experimental data^27^, was employed; the fitting coefficients were taken from ref. 28. The PMMA layer thickness was taken to be 30 nm for all core-shell systems and its dielectric constant was set to be 2.25; for simplicity CS-GNPs was were considered to be in the air. The position of a scattering cross-section peak, relevant to the dark-field spectroscopy measurements employed in the experiment, was monitored. The core radius/plasma frequency combination of 40 nm/8.6 eV resulted in 571 nm resonance, 50 nm/8.4 eV in 591 nm and 60 nm/8.2 eV in 615 nm resonances.

As the second step, the fluorescence signal from the above CS-GNP systems was determined by the integration of the time decay inputs from all the Nile Red molecules in the shell, weighted by the impact they make in the radiation into the far-field:


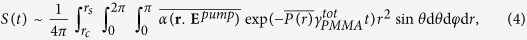
where 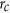 is the radius of the metal core, 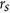 is the outer radius of the shell and the averaging is performed over all dipole moment orientations^21^. The weighting coefficients depend both on the pumping electric field at the molecule’s position and the efficiency of molecule radiation into the far field, both locally modified by the presence of the core-shell nanostructures:


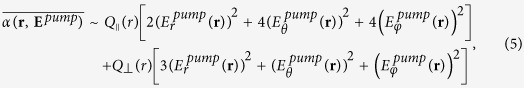
where


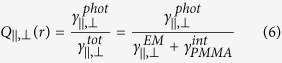
are the quantum yields of a dipole moments oriented parallel (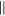) and perpendicular (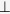) to the surface of the metallic core. 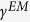 is the molecular decay rate into external electromagnetic modes which, in the core-shell case, includes photonic states (described by 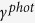) and all CS-GNP resonances (the dipole resonance being predominant due to the spectral overlap with the emitter)^24^. 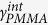 is the decay rate via internal non-radiative channels for the molecules in the PMMA environment. Using the expression for the quantum yield 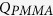 of Nile Red in PMMA:


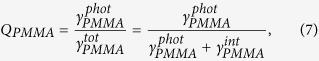
the latter can be expressed as


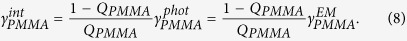


Here, it is assumed that relaxation into photons is the only external decay channel for Nile Red in bulk PMMA 
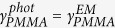
. Finally, Eq. 8 allows us to rewrite Eq. 6 and obtain the following expression for the quantum yields in the CS-GNP system:


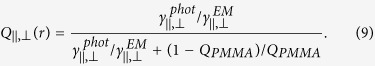


The decay times in these expressions are proportional to the powers 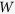 emitted by a classical radiating dipole (with a given orientation (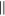 or 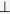) placed at the given position 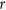) into corresponding decay channels^22^,^24^, which leads to


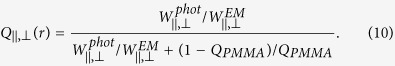


The powers in this case were determined by numerical simulations of the CS-GNP-dipole system, with the dipole oscillation frequency corresponding to the peak in Nile Red fluorescence (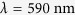), while the dipole position 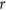 in the shell was varied. The power 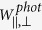 ultimately radiated to the far-field was determined via integration of the energy flow over the exterior boundary of the simulation domain. The power coupled to all electromagnetic modes of the system 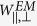, which also include the CS-GNP surface plasmon resonances, can be calculated via integration over a small sphere encircling the dipole, but located fully inside the shell domain. Alternatively, it can be found via the energy dissipation rate of the dipole^22^,^23^,^24^ :


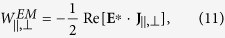
where 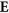 is the electric field produced by the dipole at the point of its location and 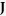 is the dipole current. Both these methods were implemented, the results showing excellent agreement. The remaining parameter in Eq. 10, 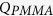 was taken to be 0.51 (Ref. 29).

The spatial distribution of the pump fields, defining the second multiplication term in the expression for the weighting coefficient (Eq. 5), was determined in a separate set of numerical simulations, in which each of the core-shell nanoparticle systems was illuminated by a plane wave with the wavelength of 514 nm.

Now we consider the Purcell factor 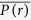 for molecules located at various positions in the shell defin the modification of their lifetime and, therefore, the time-dependent multiplication term of the integrand in Eq. 4. Averaged over all dipole orientations, 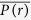 presents a linear combination of the Purcell factors corresponding to emitters with a dipole moment parallel to the metallic surface and that perpendicular to it^30^:


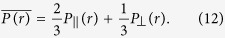
Following a derivation analogous to that for 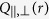, the Purcell factors may be written as


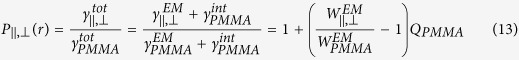
and, hence, determined from the powers 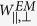 calculated above.

The last unknown term in Eq. 4 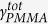 may be related to the experimentally measured reference lifetime 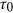 of fluorophores in a PMMA film (in regions of the sample containing no GNPs):


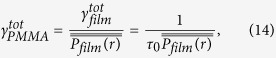
where 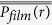 was averaged over dipole orientations (following the procedure established by Eqs 12, 13), and over various positions of the dipole across the film. The required integral power flows were determined in a separate set of numerical simulations of dipole radiation at various positions in a 30 nm thick PMMA film on a SiO_2_ substrate.

Finally, the integral in Eq. 4 was determined numerically and, to ensure consistency with the experimental procedure, for each CS-GNP configuration the obtained signal 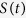 was fitted with a single exponential, corresponding to the second term in Eq. 2, returning a characteristic modified decay time 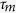 shown in Fig. 3(b).

## Additional Information

**How to cite this article**: Li, J. *et al*. Spectral variation of fluorescence lifetime near single metal nanoparticles. *Sci. Rep.* **6**, 21349; doi: 10.1038/srep21349 (2016).
