# Erratum: Spectral variation of fluorescence lifetime near single metal nanoparticles

**DOI:** 10.1038/srep22680

**Published:** 2016-03-18

**Authors:** Jia Li, Alexey V. Krasavin, Linden Webster, Paulina Segovia, Anatoly V. Zayats, David Richards

Scientific Reports 6: Article number: 2134910.1038/srep21349; published online: 02152016; updated: 03182016

The original HTML version of this Article contained a typographical error in the volume number ‘6’ which was incorrectly given as ‘5’. This has now been corrected.

